# Osteoid Osteoma: Can Impedance Levels in Radiofrequency Thermocoagulation Predict Recurrence?

**DOI:** 10.1155/2011/753502

**Published:** 2010-11-28

**Authors:** Matthew Ockendon, Jonathan J. Gregory, Gillian L. Cribb, W. Paul Cool, D. Charles Mangham, Radhesh Lalam

**Affiliations:** ^1^Department of Musculoskeletal Tumour Surgery, The Robert Jones and Agnes Hunt Orthopaedic and District Hospital, Oswestry, Shropshire SY10 7AG, UK; ^2^Department of Radiology, Robert Jones and Agnes Hunt Orthopaedic and District Hospital, Oswestry, Shropshire, SY10 7AG, UK

## Abstract

*Objective*. To evaluate rise in impedance during percutaneous radiofrequency thermocoagulation (PRFTC) of osteoid osteomas as a predictor of local recurrence. *Design and Patients*. A prospective study of 23 patients (24 PRFTC procedures) with minimum of 2.25-year followup (average 3.3 years). Average age 19.6 years (range 4–44), sex ratio 15 : 8 (male : female), 16 nondiaphyseal, 7 diaphyseal. *Results*. In 19 procedures, an increase in impedance was measured—no recurrences have occurred in this group to date. In 5 procedures, no increase in impedance was seen (3 non-diaphyseal, 2 diaphyseal), and 1 recurrence has been seen in this group to date. This difference is statistically significant with a *P* value of .05.

## 1. Introduction

Osteoid osteoma is a benign osteoblastic tumour of bone. Representing around 10% of benign bone tumours [1], these characteristically small (<2 cm) lesions have a peak incidence between childhood and early adulthood. 

Osteoid osteomas may be investigated using a number of radiographic modalities. Plain films characteristically show dense, fusiform, eccentric cortical sclerosis with a central, radiolucent “nidus.” The nidus may be obscured by the sclerosis or have undergone ossification itself.

**Figure 1:**
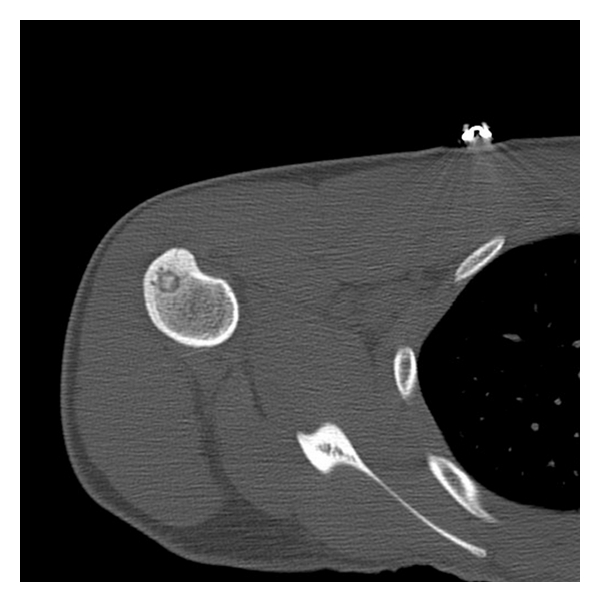
A Typical CT radiographic appearance of an osteoid osteoma in the proximal Humerus.

Tc-99 radio nucleotide bone scan may show increased uptake in the lesion. Fine slice CT scanning with bone windows is regarded as the “Gold-Standard” radiographic investigation [2] (see Figure 1). MRI has been shown to be helpful, particularly for lesions in medullary or periarticular locations and to demonstrate peritumoural oedema [3].

These lesions have been reported to undergo spontaneous regression after a number of years [4] although this is not assured and many patients find long-term treatment with nonsteroidal anti-inflammatory drugs unacceptable.

Historically the treatment of osteoid osteoma has included surgical excision; percutaneous excision under CT guidance [5], injection with ethanol [6, 7], arthroscopic debridement [8, 9], and laser photocoagulation [10, 11]. Increasingly PRFTC has become the treatment of choice. It has good reported outcomes, few side effects, short hospital stays and recurrence rates comparable with those for open surgery [12–18].

Cribb et al. showed no relationship between success of PRFTC and patient age, duration of symptoms, size of lesion or previous treatment. They did note, however, that nondiaphyseal lesions were more likely to recur than those in the diaphysis [19]. 

We measured impedance during PRFTC and assessed it as a predictor of success in the treatment of osteoid osteoma. Impedance is the measure of opposition to flow of an alternating current (AC), analogous to “resistance” in a direct current (DC) system. It is expressed as a voltage-current ratio for a given frequency. 

Impedance rises during PRFTC as proteins are de-natured, and the area immediately adjacent to the probe is desiccated. Impedance can be calculated in real time by the radiofrequency generator.

## 2. Materials and Methods

23 patients with osteoid osteoma were treated by CT-guided PRFTC at the Robert Jones and Agnes Hunt Orthopaedic and District Hospital, Oswestry. There was a male preponderance with 15 male and 8 female patients. All patients were investigated with plain radiographs and CT. In some patients MRI scanning and/or radionucleotide bone scanning was also performed. The diagnosis was made by a specialist multidisciplinary team including musculoskeletal radiologists, orthopaedic oncological surgeons, and musculoskeletal pathologists. Histological confirmation of the diagnosis of osteoid osteoma is not thought to be necessary owing to the diagnostic specificity of the clinical and radiological features [20]. Patients in whom there was debate about the diagnosis were excluded from this study.

Of the 23 osteoid osteomas treated, 16 were nondiaphyseal, and 7 were diaphyseal.

The PRFTC procedures were performed in the CT scanning suite (Figure 3), under general anaesthetic, by a consultant musculoskeletal radiologist and a consultant orthopaedic oncological surgeon. The lesions were located by fine (3 mm) cuts. A Bonopty needle (Radi Medical Systems, Uppsala, Sweden) was inserted under CT guidance into the lesion to take biopsies for histological examination and microbiological cultures. The radiofrequency electrode (RITA Starburst—RITA Medical Systems, Mountain View, Calif. USA) was placed within the lesion under CT control. A radiofrequency generator (RITA generator, RITA Medical Systems Atlanta, GA, USA) was then used to raise the temperature of the lesion to 90°C for 4 minutes in each case. For lesions in which the nidus was smaller than 1 cm, a single electrode position was used, for larger lesions multiple needle positions were used—sometimes utilising a separate approach.

All patients were allowed home the day following the procedure with no restrictions on activity or weight bearing. Followup was for a mean of 3.3 years (range 2.25–4.87 years).

Followup was clinical, and symptomatic patients underwent a further cycle of radiological investigation. Asymptomatic patients did not undergo further radiological investigation.

### 2.1. Statistical Methods

Univariate statistical analysis was performed using Satview for Windows (version 5, SAS Institute). The Chi-Squared test was used to compare recurrence rates between the groups with and without a rise in impedance.

## 3. Results

In 18 procedures an increase in impedance was measured—no recurrences have occurred in this group to date. In 5 procedures no increase in impedance was seen (3 nondiaphyseal, 2 diaphyseal).

**Figure 2:**
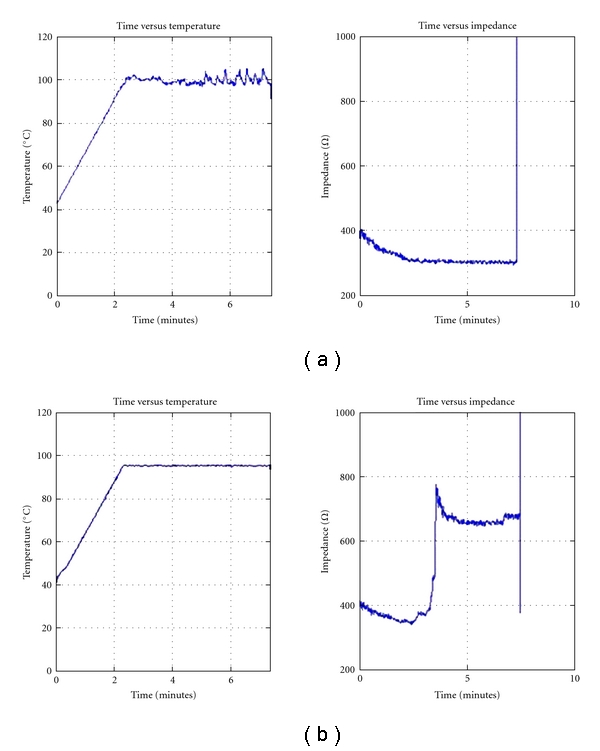
Graphs of probe temperature and impedance against time (a) with no observed rise in impedance and (b) same patient, repeat procedure with observed rise in impedance.

**Figure 3:**
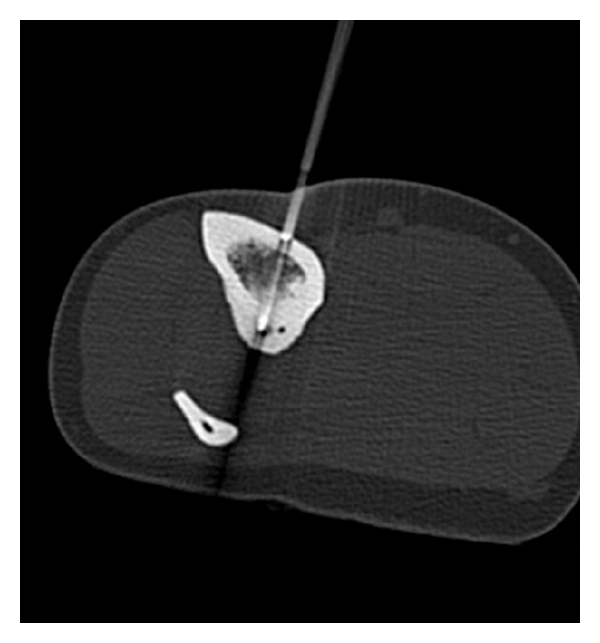
Positioning the radiofrequency probe under CT guidance.

There was a single local recurrence. This was in a nondiaphyseal lesion in which no rise in impedance had been measured. This occurred 11 months after the PRFTC. A second attempt at PRFTC was successful. At the second procedure an increase in impedance was observed (see Figure 2).

The success rate in the group in which an increase in impedance was measured was 100% and in the group in which no increase in impedance was measured was 80% (4 out of 5).

Chi Squared test between the groups with and without an observed impedance rise (for incidence of recurrence) shows a statistically significant difference with a *P* value of .05.

The average increase in impedance for diaphyseal lesions was nearly twice that observed for nondiaphyseal lesions.

## 4. Discussion

Numerous studies exist showing excellent results for the treatment of Osteoid Osteoma with Percutaneous Radiofrequency Ablation [12–18]. It is widely regarded as the “Gold-Standard” for the treatment of these lesions although reported success from the primary procedure varies between 77 and 100% [21]. 

Our overall success rate, in this study, was 96% (23 out of 24). This fell to 80% (4 out of 5) in those procedures where no rise in impedance was observed.

To date the only factor predicting outcome from PRFTC has been anatomical location (diaphyseal versus metaphyseal) [16, 19], and no literature exists, to our knowledge, exploring rise in impedance as a predictive factor (Table 1). 

We found a significantly greater rise in impedance during the procedure in those lesions located in the diaphysis of a bone. We postulate that such lesions are often better “contained” by the surrounding cortex and cortical reaction. Furthermore, diaphyseal lesions are likely to have a less rich blood supply compared to those in the metaphysis. It is likely, therefore, that the metaphyseal locations have significant cooling effects from circulating blood reducing the efficacy of the treatment. Although the probe tip temperature (as controlled by the radiofrequency generator) will have been consistent, the gradient of temperature away from the probe may be steeper. The diminished change in impedance therefore represents a smaller treated area, and this may help to explain the higher risk of recurrence in nondiaphyseal locations.

We find it useful to monitor impedance during PRFTC of osteoid osteomas. Impedance is calculated in real time by the majority of radiofrequency generators, and we propose its routine use. Where a rise in impedance is not achieved, repositioning of the electrode and additional cycles of treatment may be indicated.

## Figures and Tables

**Table 1:** Impedance change during PRFTC procedures according to anatomical location.

	Average start impedance (*Ω*)	Average end impedance (*Ω*)	Average increase (*Ω*)	Average % increase
Overall	345	493	173	58%
Nondiaphyseal	321	412	203	37%
Diaphyseal	477	524	113	65%
